# Inequality of Experience of Dental Caries between Different Ethnic Groups of Brazilians Aged 15 to 19 Years

**DOI:** 10.1371/journal.pone.0145553

**Published:** 2015-12-22

**Authors:** Andreia Maria Araújo Drummond, Efigênia Ferreira Ferreira, Viviane Elisangela Gomes, Wagner Marcenes

**Affiliations:** 1 Institute of Dentistry, Barts and The London School of Medicine and Dentistry, Queen Mary University of London. London, United Kingdom; 2 Faculty of Dentistry, Universidade Federal de Minas Gerais. Belo Horizonte, Minas Gerais, Brazil; Navoadaya Dental College and Hospital, mantralayam Road, INDIA

## Abstract

**Introduction:**

The aim of this study was to assess inequality of experience of dental caries, based on race/ethnicity, among Brazilian adolescents aged 15 to 19 years in 2010 and test whether socioeconomic indicators fully explain ethnic differences in dental caries.

**Methods:**

Data from a National Oral Health Survey conducted in Brazil in 2010 was analysed. Race/ethnicity was self-assigned and modified to White, African descents, East Asian descents, Mixed Race and Indigenous descents. The prevalence of caries experience by race/ethnic group in 2010(n = 5,367) was calculated. Further analysis included conceptual hierarchical modelling and mediation analysis.

**Results:**

Caries experience was 76.9% in 15 to 19 year old Brazilians in 2010. While African descents were 32% more likely to have caries experience than Whites, Mixed Race were 69% more likely to have caries experience than Whites. Hierarchical conceptual modelling analysis confirmed the highly significant association between caries and race/ethnicity. Mixed Race and East Asian descents were 1.44 (95% CI 1.24–1.67) and 1.81 (95% CI 1.02–3.20) times more likely to experience caries than Whites after adjusting for age, sex, education and income. The difference in the likelihood of experiencing caries between Whites and African descents was not statistically significant after adjusting for years of education and family income. The results of mediation analysis confirmed that inequality of caries experience between Whites and Mixed Race and East Asian descents was mediated through education and income. The likelihood that Mixed Race and East Asian descents would experience caries compared to Whites was attenuated, by 14.8% and by 9.5% respectively, after adjusting for years of education and income.

**Conclusions:**

Data analysis demonstrated that Whites have benefited more from the significant reduction in dental caries experience in 15 to 19 year old Brazilians, as compared to African descents and Mixed Race. Education and income fully explained ethnic inequalities in experience of dental caries between Whites and African descents, and largely explained inequalities between Whites and Mixed Race.

## Introduction

The literature showed consistently that socially vulnerable groups had a higher prevalence of experience of dental caries [1–17]. It has been suggested in the literature that the race and ethnicity of a person may determine their oral health status [2, 12]. There is a considerable amount of literature on the utility of race and ethnicity as concepts [18–20]. ‘Race’ and ‘ethnicity’ are terms commonly used, with similar characteristics but subtle differences. ‘Race’ typically refers to physical features, such as skin colour or hair texture, which may reflect a person’s ancestry; and examples of categories that are commonly used to describe race include White, African descents, or Asian descents. ‘Ethnicity’ is a complex term used to describe perceived social groupings based on a sense of belonging, place of origin, and other factors such as language, religion, and sometimes race [19, 21]. Assessing ethnicity is difficult, and numerous proxy measures are used to capture its various components. For the purposes of this study, ‘Ethnicity’ and ‘Race’ will be viewed as synonyms. Self-reported ethnicity captures both the shared experiences/culture of an individual and their self-identity [20, 21].

The association between experience of dental caries and race/ethnicity is not well-defined. Worldwide, White children, adolescents, adults and the elderly were consistently found to experience lower levels of dental caries than their counterparts from other racial/ethnic groups [1–17]. However, it is not clear whether these differences were due to race/ethnicity *per se* or due to confounding variables such as socio-economic position (“SEP”) as both are strongly related to experience of dental caries [1–3, 5, 6, 8, 14–17, 22–24]. Data from the United States suggested that SEP may fully explain ethnic inequalities in oral health [25, 26], while other studies have reported the persistence of these inequalities after adjustment for confounding factors [15, 27].

The aim of this study was to assess inequality of experience of dental caries as between different ethnic groups of Brazilian adolescents (aged 15 to 19 years) in 2010. In addition, the study sought to test whether socioeconomic indicators fully explain ethnic differences in dental caries in 2010.

## Methods

This article analyses data from a National Oral Health Survey conducted in Brazil in 2010 (SBBrasil 2010) [28]. The survey adopted a cross-sectional survey design and included a representative sample of young adults aged 15 to 19 years. Participants were selected using multi-stage random sampling stratified approach by the five Brazilian macro-regions. Proportional representation was adopted. Trained and calibrated dentists conducted the oral examinations using the WHO protocol [29]. The DMFT index was used to assess experience of dental caries. Following the clinical examination, participants answered a supervised structured questionnaire, designed especially for the research. A handheld Personal Digital Assistant (PDA model P550) was used for data collection. The questionnaire included questions on socio-demographic factors (age, sex, race/ethnicity, years of education, family income and household characteristics). Age was assessed in complete years. Race/ethnicity was self-assigned into the five Brazilian census classification [30] and modified to White, African descent, East Asian descent, Mixed Race (those with mixed racial ancestry, known as “*pardos*”) and Indigenous descent. The participant’s ‘years of education’ were assessed in terms of the number of successfully completed school years; and ‘family income’ was reported by the head of the family, considering the total income of the household in the month prior to the interview. Detailed information on the sampling procedure can be found elsewhere [31]. The SBBrasil 2010 (*Conselho Nacional de Etica em Pesquisa*—approval ref. 15,498/2010) followed the requirements of the Declaration of Helsinki and was approved by the Brazilian Ethics Committee.

### Statistical analysis

Data was analyzed using IBM SPSS Statistics 18.0. The prevalence of experience of dental caries was calculated by reference to race/ethnic group. The SBBrasil 2010 included a sample of 5,445 participants, aged 15 to 19 years. Seventy-eight individuals (1.43%) were excluded from this data analysis due to missing information on the outcome variable (n = 5,367).

Further data analysis were carried out to assess race/ethnicity inequalities of experience of dental caries. In addition, to test the estimation of precision, we also performed post hoc power analysis. The post-hoc sample calculation demonstrated that the minimum sample size needed to provide 80% statistical power to enable the identification of an odds ratio of 1.5 was estimated to be 822 [32]. The calculation assumed 50% of the unexposed population and 60% of the exposed population to have the outcome of interest: α equal to 0.05, and β equal to 0.20. Therefore, the sample size (n = 5,367) provides sufficient statistical power to test the hypothesis.

Data manipulation was minimal. The participants’ ‘years of education’ were categorized into ‘four or less years of education’, ‘five to eight years of education’ and ‘nine or more years of education’. Family income’ was converted into U.S. dollars (US$1.00 = R$1.76 in 2010) and categorized into ‘equal to or less than US$142.00’, ‘US$143.00 to US$284.00’, ‘US$285.00 to US$852.00’, ‘US$853.00 to US$1,420.00’ and ‘US$1,421.00 or above’.

Simple logistic regression analyses were carried out to assess the unadjusted association between each of the explanatory variables (race/ethnicity, sex, age, years of education and family income) and experience of dental caries. In accordance with the lax criterion [33], explanatory variables that were not statistically significantly related to the outcome at the level of 0.20 or more were excluded at this stage. Following this, conceptual hierarchical modelling [34] was carried out. The remaining variables were sequentially included as follows: (1) race/ethnicity, sex and age (2) race/ethnicity, sex, age plus years of education; (3) race/ethnicity, sex, age, years of education, plus family income. Odds Ratios (OR) were reported and the 95% confidence interval was considered. Attenuation of the OR was calculated using the formula (ORU-ORA)÷(ORU-1) [35], where ORU represents the odds ratio before including the family income and ORA reflects the odds ratio after including years of education and family income in the model. Mediation analysis included the four steps proposed by Baron and Kenny [36].

## Results

Frequency distribution of demographic characteristics in the SBBrasil 2010 study sample are shown in Table 1. Experience of dental caries was 76.9% in 15 to 19 year old Brazilians in 2010. A significant difference in the distribution of experience of dental caries among race/ethnic groups was observed. African descents (77.1%), East Asian descents (83.5%), Mixed Race (81.0%) experienced significantly more dental caries than Whites (71.9%) while experience of dental caries among Indigenous descents (75.0%) was not significantly different than among Whites (Table 2). In addition, there were statistically significant differences in the distribution of experience of dental caries by age, sex, years of education and family income. As expected, experience of dental caries increased with ageing. Participants with less than four years of education were 2.67 (95% CI 1.72–4.15) times more likely to have experience of dental caries than those with nine or more years of education. Participants with family income of US$142.00 or less were 3.52 (95% CI 2.20–5.63) times more likely to have experience of dental caries than those with a family income of US$ 1,421.00 or above (Table 2).

**Table 1:** Sample characteristics by race/ethnicity in 15 to 19 years old Brazilians in 2010.

Characteristics		Sample (n = 5367)	White (n = 2177; 40.6%)	African descents (n = 586; 10.9%)	East Asian descents (n = 103; 1.9%)	Mixed Race (n = 2453; 45.7%)	Indigenous descents (n = 48; 0.9%)
Age							
	15 years	1438 (26.8%)	552 (25.4%)	167 (28.5%)	29 (28.1%)	672 (27.4%)	18 (37.5%)
	16 years	973 (18.1%)	404 (18.6%)	85 (14.5%)	18 (17.5%)	458 (18.7%)	8 (16.7%)
	17 years	977 (18.2%)	392 (18.0%)	100 (17.1%)	25 (24.3%)	451 (18.4%)	9 (18.7%)
	18 years	995 (18.6%)	419 (19.2%)	110 (18.8%)	14 (13.6%)	444 (18.1%)	8 (16.7%)
	19 years	984 (18.3%)	410 (18.8%)	124 (21.1%)	17 (16.5%)	428 (17.4%)	5 (10.4%)
Gender							
	Male	2452 (45.7%)	1014 (46.6%)	265 (45.2%)	43 (41.7%)	1101 (44.9%)	29 (60.4%)
	Female	2915 (54.3%)	1163 (53.4%)	321 (54.8%)	60 (58.3%)	1352 (55.1%)	19 (59.6%)
Education							
	≤ 4 years	200 (3.7%)	56 (2.6%)	34 (5.8%)	4 (3.9%)	103 (4.2%)	3 (6.2%)
	5–8 years	1798 (33.5%)	618 (28.4%)	236 (40.3%)	24 (23.3%)	901 (36.7%)	19 (39.6%)
	≥ 9 years	3357 (62.6%)	1498 (68.8%)	313 (53.4%)	75 (72.8%)	1445 (58.9%)	26 (54.2%)
	*Missing information*	*12* (0.2%)	*5* (0.2%)	*3* (0.5%)	*0* (0%)	*4* (0.2%)	*0* (0%)
Family Income							
	≤ US$142	160 (3.0%)	36 (1.7%)	26 (4.4%)	4 (3.9%)	91 (3.7%)	3 (6.2%)
	US$143–284	686 (12.8%)	203 (9.3%)	104 (17.8%)	14 (13.6%)	359 (14.6%)	6 (12.5%)
	US$285–852	2621 (48.8%)	943 (43.3%)	307 (52.4%)	46 (44.7%)	1302 (53.1%)	23 (47.9%)
	US$853–1420	920 (17.1%)	456 (20.9%)	87 (14.8%)	16 (15.5%)	352 (14.3%)	9 (18.8%)
	≥ US$1421	665 (12.4%)	406 (18.7%)	37 (6.3%)	10 (9.7%)	210 (8.6%)	2 (4.2%)
	*Missing information*	*315* (5.9%)	*133* (6.10%)	*25* (4.3%)	*13* (12.6%)	*139* (5.7%)	*5* (10.4%)
DMFT							
	= 0 (free of caries)	1240 (23.1%)	611 (28.1%)	134 (22.9%)	17 (16.5%)	466 (19.0%)	12 (25.0%)
	≥ 1	4127 (76.9%)	1566 (71.9%)	452 (77.1%)	86 (83.5%)	1987 (81.0%)	36 (75.0%)

**Table 2:** Caries experience (DMFT>0) by demographic and socioeconomic characteristics in the total sample, Brazil, 2010.

Characteristics		N DMFT>0 (%)	OR^a^ [95%CI]	*P value*
Self-reported race/ethnicity				
	White	1566 (71.9)	1 [reference]	
	African descents	452 (77.1)	1.32 [1.06–1.63]	*0*.*012*
	East Asian descents	86 (83.5)	1.97 [1.16–3.35]	*0*.*012*
	Mixed Race	1987(81.0)	1.66 [1.45–1.91]	*<0*.*001*
	Indigenous descents	36 (75.0)	1.17 [0.60–2.26]	*0*.*640*
Sex				
	Male	1854 (75.6)	1 [reference]	
	Female	2273 (78.0)	1.14 [1.01–1.30]	*0*.*041*
Age				
	15 years	1029 (71.6)	1 [reference]	
	16 years	735 (75.5)	1.23 [1.01–1.48]	*0*.*031*
	17 years	771 (78.9)	1.49 [1.23–1.80]	*<0*.*001*
	18 years	793 (79.7)	1.56 [1.29–1.89]	*<0*.*001*
	19 years	799 (81.2)	1.72 [1.41–2.09]	*<0*.*001*
Education				
	≤ 4 years	177 (88.5)	2.67 [1.72–4.15]	*<0*.*001*
	5–8 years	1448 (80.5)	1.44 [1.25–1.65]	*<0*.*001*
	≥ 9 years	2492 (74.3)	1 [reference]	
	*Missing information*	*10 (83*.*3)*		
Family Income				
	≤ US$142	137 (85.6)	3.52 [2.20–5.63]	*<0*.*001*
	US$143–284	571 (83.2)	2.93 [2.27–3.79]	*<0*.*001*
	US$285–852	2090 (79.7)	2.33 [1.94–2.78]	*<0*.*001*
	US$853–1420	678 (73.7)	1.66 [1.34–2.05]	*<0*.*001*
	≥ US$1421	418 (62.9)	1 [reference]	
	*Missing information*	*233 (74*.*0)*		

^a^Unadjusted logistic regression models and odds ratios (OR) reported with a 95% confidence interval

Hierarchical conceptual modelling analysis (Table 3) confirmed the highly significant association between dental caries and race/ethnicity. Adjusted odds ratios confirmed that Mixed Race and East Asian descents were 1.44 (95% CI 1.24–1.67) and 1.81 (95% CI 1.02–3.20) times more likely to experience dental caries than Whites respectively. The difference in the likelihood of experiencing dental caries between Whites and African descents was not statistically significant after adjusting for years of education and family income.

**Table 3:** Models for race/ethnic differences in caries experience (DMFT) among the total sample, Brazil, 2010.

Self-reported race/ethnic groups	Model 1^a^	*P value*	Model 2^a^	*P value*	Model 3^a^	*P value*
	OR^b^ (95% CI)		OR^b^ (95% CI)		OR^b^ (95% CI)	
White	1.00 [Reference]		1.00 [Reference]		1.00 [Reference]	
African descents	1.32 [1.07–1.64]	*0*.*011*	1.21 [0.97–1.51]	*0*.*086*	1.04 [0.83–1.30]	*0*.*762*
East Asian descents	2.00 [1.18–3.41]	*0*.*010*	2.04 [1.20–3.46]	*0*.*009*	1.81 [1.02–3.20]	*0*.*042*
Mixed Race	1.69 [1.47–1.94]	*<0*.*001*	1.61 [1.40–1.86]	*<0*.*001*	1.44 [1.24–1.67]	*<0*.*001*

^a^ Model 1 was adjusted for sex and age; Model 2 was adjusted for sex, age plus years of education; Model 3 was adjusted for sex, age, years of education plus family income.

^b^ Logistic regression models were fitted and odds ratios (OR) reported.

The results of mediation analysis, using the four steps proposed by Baron and Kenny [36] confirmed that race/ethnic inequalities of experience of dental caries between Whites and Mixed Race and East Asian descents was mediated through education and income. Race/Ethnicity was significantly associated with experience of dental caries, education and income. Also, the likelihood that Mixed Race and East Asian descents would experience dental caries compared to Whites was attenuated by 14.8% and by 9.5% respectively after adjusting for years of education and income. Table 3 shows all models compared to the unadjusted model and, interestingly, education and income fully explained the difference in experience of dental caries between Whites and African descents.

## Discussion

The unadjusted results of this study corroborated previous studies worldwide that postulated that Whites experience significantly less dental caries than other race/ethnic groups [1–17]. Wu et al. [17] also conducted a hierarchical analysis and their results corroborated our findings. The findings also confirmed the well-established association between experience of dental caries and income and education worldwide [23] and in the Brazilian population [24]. To our knowledge, this is the only study that has conducted mediation analysis.

Mediation analysis demonstrated that higher education and income may explain inequality of experience of dental caries between race/ethnic groups in Brazil. While education and income fully accounted for the difference in experience of dental caries between Whites and African descents, the likelihood that Mixed Race and East Asian descents would experience dental caries compared to Whites was significantly attenuated.

Although experience of dental caries has reduced significantly among all race/ethnic groups in 15 to 19 year old Brazilians between 2003 and 2010, inequality of experience of dental caries between these groups has increased. Experience of dental caries has reduced significantly from 89.2% to 76.9% in 15 to 19 year old Brazilians between 2003 [37] and 2010 [28]. Comparison of the results of the national studies carried out in Brazil in 2003 and 2010 showed a larger reduction in prevalence of experience of dental caries in Whites (19.4%) than in Indigenous descents (13.8%), African descents (11.2%), Mixed Race (9.7%) and East Asian descents (7.2%). Furthermore, while in 2003 African descents were 19% less likely to have experience of dental caries than Whites, in 2010 African descents were 32% more likely to have experience of dental caries than Whites. Also, while in 2003 Mixed Race and Whites had similar likelihood of experiencing caries, in 2010 Mixed Race were 69% more likely to have experience of dental caries than Whites (results not presented).

In the past 10 years, the Brazilian government have been investing in health, education and income policies to reduce inequality. A Family Health Programme (FHP), which aimed to broaden access to public health services, especially in deprived areas, was introduced in Brazil in 1994. The FHP covered 94.3% of Brazilian municipalities and 50.8% of the population in 2010. The FHP offers free community based primary care, and follows the principles of decentralisation, universality and equity [38]. In 2003, the “*Bolsa Família*” (Family Allowance) programme was introduced, which is a major cash transfer programme targeting those in poverty. It aimed to promote an immediate relief of poverty, improving access to education and health care, and offering complementary social programmes to enable families to end their condition of vulnerability. Enrolment in the “*Bolsa Família*” programme is conditional on school attendance up to 17 years and meeting primary health care conditions such as vaccination and nutritional surveillance [39].

In 2004, positive discrimination or social inclusion policies established racial quotas reserving 20% of available admissions in universities for students who self-identified as African descents [40]. However, Whites may have benefited more from the Brazilian economic development and social programmes than other groups or the programmes were still insufficient to reduce inequalities between the race/ethnic groups in Brazil.

Some limitations of this study need to be discussed. Measuring race/ethnicity in Brazil is challenging [18]. The aggregation of racial categories is necessary because of the wide range of mixed races from “darker to lighter Mixed Race”. This may lead to misclassification as light Mixed Race may classify themselves as Whites and dark Mixed Race as African descents. Brazilian government policies to reduce ethnic inequality includes positive discrimination, i.e. university entry quotas for African descents and Mixed Race [40]. This may have shifted the way Brazilians have self-assessed their race/ethnicity [41]. East Asian descents and Indigenous populations are minority groups in the Brazilian population leading to a smaller sample than Whites, African descents and Mixed Race, which led to larger confidence intervals. While classifying participants into Whites and non-Whites may reduce misclassification it obscures the relevant differences. The cross-sectional design precludes establishing a cause-effect relationship. However, it is more logical to assume that race/ethnicity determines experience of dental caries than the reverse. Race/ethnicity is established at birth; and caries in the permanent dentition starts at age of 6 years. In addition, there is not any clear evidence of racial or genetic variations as determining factors of experience of dental caries. On the contrary, there is strong evidence of ethnic differences in relation to exposure to the risk factors associated with experience of dental caries.

In conclusion, data analysis of the SBBrasil 2010 demonstrated that Whites have benefited more from the significant reduction in experience of dental caries in 15 to 19 year old Brazilians, as compared with African descents and Mixed Race. Education and income fully explained ethnic inequalities in experience of dental caries between Whites and African descents, and largely explained inequalities between Whites and Mixed Race.
